# Host Plant-Based Artificial Diets Enhance Development, Survival and Fecundity of the Edible Long-Horned Grasshopper *Ruspolia differens* (Orthoptera: Tettigoniidae)

**DOI:** 10.1093/jisesa/ieac003

**Published:** 2022-03-29

**Authors:** Alfonce Leonard, James P Egonyu, Chrysantus M Tanga, Samuel Kyamanywa, Sunday Ekesi, Fathiya M Khamis, Sevgan Subramanian

**Affiliations:** 1 Environmental Health Theme, International Centre of Insect Physiology and Ecology, P.O. Box 30772-00100, Nairobi, Kenya; 2 Department of Agricultural Production, Makerere University, P.O. Box 7062, Kampala, Uganda; 3 Department of Crops, Tanzania Agricultural Research Institute (TARI)-Ukiriguru, P.O. Box.1433, Mwanza, Tanzania

**Keywords:** mass rearing, diet formulation, development time, mortality, fecundity

## Abstract

Wild swarms of the long-horned grasshoppers *Ruspolia differens* (Serville) which are widely harvested for consumption and sale in Africa are seasonal and unsustainable, hence the need for innovative ways of artificially producing the insects. We investigated the development, survival, and reproduction of *R. differens* in the laboratory on diets mixed with host plants [*Digitaria gayana* Kunth, *Cynodon dactylon* (L.) and *Megathyrsus maximus* Jacq (Poales: Poaceae); *Ageratum conyzoides* L. (Asterales: Asteraceae)] identified from guts of their wild conspecifics with a view to developing a suitable diet for artificial mass rearing of the edible insect. A standard diet comprising ground black soldier fly, *Hermetia illucens* L. (Diptera: Startiomyidae) larvae, soybean flour, maize flour, vitamin premix, and ground bones was tested for rearing *R. differens* as a control against the same ingredients incorporated with individual powders of the different host plants. Whereas *R. differens* developed more slowly in the diet mixed with *D. gayana* than in the control diet; its development was faster in the diet mixed with *C. dactylon*. Mortalities of *R. differens* in host plant-based diets were 42.5–52.5%, far lower than in the control diet with 71% mortality. The insects raised on the diet mixed with *M. maximus* laid approximately twice more eggs compared to *R. differens* fecundities from the rest of the diets. However, inclusion of host plants in the diets had no detectable influence on *R. differens* adult weight and longevity. These findings support inclusion of specific host plants in artificial diets used for mass rearing of *R. differens* to enhance its survival, development, and fecundity.

The long-horned grasshopper *Ruspolia differens* (Serville) is an important edible insect in Eastern and Southern Africa (Agea et al. 2008, Kinyuru et al. 2010, Valtonen et al. 2018). *R. differens* has substantial nutritional value for humans as it contains 43–44% protein, 46–48% fat, 3% ash, and 4–5% crude fiber (Kinyuru et al. 2010). These insects are composed of essential minerals such as phosphorous (426–673 mg/100 g), potassium (446–673 mg/100 g), calcium (34.9–128 mg/100 g), iron (8.0–116.0 mg/100 g), and zinc (5.7–12.9 mg/100 g) (Kinyuru et al. 2018, Ssepuuya et al. 2019). They also contain vitamins such as A (0.69–2.8 μg/g), E (135.9–201.0 μg/g), B3 (0.12–3.30 mg/100 g), B2 (0.84–2.80 mg/100 g), C (0.01–3.01 mg/100 g), B12 (0.73–1.35 μg/100 g), B9 (0.10–0.90 mg/100 g) and B6 (0.14–0.41 mg/100 g) (Kinyuru et al. 2018, Ssepuuya et al. 2019). *R. differens* is also composed of essential amino acids like histidine (15.0–51.4 mg/g), isoleucine (30.0–51.1 mg/g), leucine (59.0–78.3 mg/g), lysine (45.0–54.2 mg/g), methionine (16.0–27.5 mg/g), cysteine (6.0–18 mg/g), threonine (23.0–35.0 mg/g), tryptophane (6.0–14.3 mg/g) and valine (39.0–73.3 mg/g) (Kinyuru et al. 2018). Owing to their distant relationship with humans, grasshoppers have minimum risk of transmitting zoonotic diseases compared to livestock (Paul et al. 2016). Besides being a source of healthy food, *R. differens* provides employment for improved livelihoods in both rural and urban areas (Agea et al. 2008). In 2008, *R. differens* had a value of US$ 2.8 per kg in Kampala, a price which was 40% higher than that of beef (Agea et al. 2008).

*R. differens* occurs in swarming and nonswarming phases in tropical Africa and some Indian Ocean islands (Bailey and McCrea 1978, Massa 2015, Opoke et al. 2019). Swarms occur during peak rains, for instance around April–May and in November–December in Uganda (Bailey and McCrea 1978, Opoke et al. 2019). Wild swarms of *R. differens* are commercially harvested using light traps during swarming seasons only (Mmari et al. 2017, Okia et al. 2017, Opoke et al. 2019).

Efforts have been intensified to develop protocols for mass rearing as an alternative to wild harvests of *R. differens,* especially during nonswarming phase (Malinga et al. 2018a, Malinga et al. 2018b, Lehtovaara et al. 2018, Lehtovaara et al. 2019). Previous attempts to develop diets for artificial rearing of *R. differens* relied on testing acceptance of plants on which the insects roost in the wild and other ingredients like dog biscuits, dried blood, lucerne meal, Pro-Nutro (a well-balanced protein), Chicken super feed egg buster and simsim cake (Brits and Thornton 1981, Malinga et al. 2018a). *R. differens* have been reared under temperatures ranging from 25 to 30°C and relative humidity of 50–73% (Hartley 1967, Brits and Thornton 1981, Malinga et al. 2018a, Valtonen et al. 2018). These efforts have yielded low survival rate of about 38% (Malinga et al. 2018a), which may be attributable to lack of important plant nutrients (Behmer and Joern 1993). Limited knowledge on natural host plants of *R. differens* could be a hindrance to successive captive rearing of the insect (Rutaro et al. 2018, Opoke et al. 2019).

Both immature and adult *R. differens* have strong chewing mouth parts that enable them to feed on a variety of food (Matojo and Hosea 2013). Although these insects are polyphagous in the wild, they prefer feeding on grasses to other plants (Valtonen et al. 2018, Opoke et al. 2019, Leonard et al. 2020). *R. differens* selectively feeds on specific plant parts, preferring seeds and flowers followed by stems and leaves (Hartley 1967, Valtonen et al. 2018). Its preference for seeds and flowers could be attributed to higher protein content in these plant parts compared to stems and leaves (Opoke et al. 2019). Nutrient levels such as proteins, carbohydrates, and minerals in plants influence survival, development, growth, and reproduction of grasshoppers (Nayar 1964, Joern and Behmer 1998). Grasshoppers can maintain the required nutrient levels in their body tissues by manipulating the digestion time of ingested plants or by selecting plants with high-quality proteins (Joern and Behmer 1998, Cammack and Tomberlin 2017). At nymphal stage, grasshoppers require almost 1:1 ratio of digestible carbohydrates and proteins (Joern and Behmer 1998). Nutrient levels of 4% proteins and 15–26.7% carbohydrate in diets have been reported to increase egg production in short-horned grasshoppers *Melanoplus sanguinipes* (Fabricius) (Orthoptera: Acrididae) and *Phoetaliotes nebrascensis* (Thomas) (Orthoptera: Acrididae) (Joern and Behmer 1997, Joern and Behmer 1998).

Natural host plants have essential amino acids such as phenylalanine which is important for cuticle formation in insects for enhanced survival (Behmer and Joern 1993). These amino acids and sterols concentration in host plants also enhance fecundity in insects (Awmack and Leather 2002). Opoke et al. (2019) observed *R. differens* roosting on 19 grasses and 2 sedges during nonswarming phase, and considered these as its host plants. Leonard et al. (2020), analyzed plant materials from the guts of swarming *R. differens* using molecular tools and found that they were dominated by species in Poaceae family, mostly *Digitaria gayana* Kunth (Poales: Poaceae). Other plant families identified in *R. differens* guts were Fabaceae, Asteraceae, Myrtaceae, Polygonaceae, and Rutaceae. It is however foolhardy to assume that what the insects naturally feed on provides the best results in mass rearing (Lattanzio et al. 2006). The performance of *R. differens* on diets incorporated with their identified host plants, therefore, needs to be investigated.

Diet formulation for phytophagous insects require components like B-vitamins, amino acids, lipids, organic ions, carbohydrates, and water (Nayar 1964). These diet components can be mixed with several plants to improve the performance of insects (Bernays et al. 1994, Singer and Bernays 2003, Malinga et al. 2018a, Malinga et al. 2018b, Opoke et al. 2019). A high proportion of protein is required in grasshopper diets for growth and development (Nayar 1964). Black soldier fly larvae with up to 50% crude protein (Tschirner and Simon 2015, Shumo et al. 2019), or soybeans (48%) (Yamka et al. 2003), are good sources of protein for insect rearing. Many grass plants have low dry matter protein of up to 2% (Berner et al. 2005). Therefore, evaluation of the performance of *R. differens* on known artificial diets mixed with host plants identified from its gut was warranted.

This study aimed at investigating the performance of *R. differens* on diets incorporated with different host plants for improving their artificial mass rearing. We hypothesized that incorporation of host plants into artificial diet influences development time, mortality, adult longevity, fecundity, and adult weight of *R. differens*.

## Materials and Methods

### Colony Initiation

**Fig. 1.**
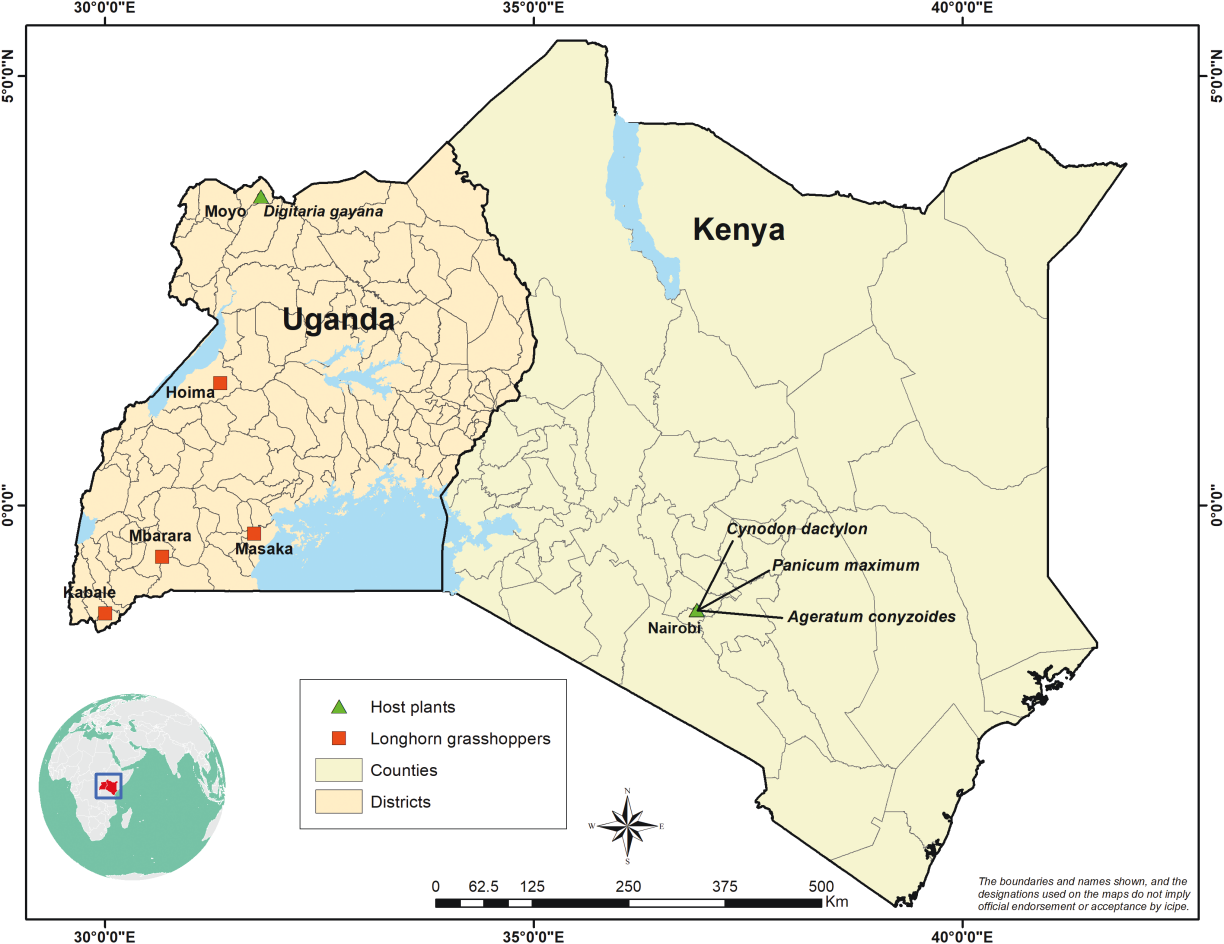
Insects and host plant collection sites in Uganda and Kenya.

The nymphs of *R. differens* for this study were obtained from a laboratory colony at *icipe*. The colony was established with about 1,000 *R. differens* adults collected in November 2018 in Uganda. Locally available plants were selected from identified host plant species of *R. differens* (Leonard et al. 2020) and from Opoke et al. (2019). Selected plants were *D. gayana*, *Cynodon dactylon* L. (Poales: Poaceae), *Ageratum conyzoides* L. (Asterales: Asteraceae), and *Megathyrsus maximus* Jacq. (Poales: Poaceae). Apart from *D. gayana*, which was collected in Moyo, in northern Uganda, experimental host plants were collected at *icipe*, Nairobi, Kenya. The GPS data for *R. differens* and host plants collection sites were subjected to QGIS to construct a map (Fig. 1).

### Preparation of Host Plants

The plant materials were weighed and dried at 65°C in a dry heat oven (Model VT6130M, Heraeus Instrument Vacutherm, Germany). The weights before drying were 280 g, 150 g, 150 g, and 144 g; and after drying were 43.12 g, 42.69 g, 39.98 g, and 33.70 g for *A. conyzoides, C. dactylon, D. gayana,* and *M. maximus*, respectively. Dried plants were ground using a mixer grinder (Preethi Trio, India).

### Diet Formulation and Experimental Design

In the preliminary study on development and survival of *R. differens,* single ground dry host plant diets showed that the insects could not survive beyond 3 d for all four plants. Hence, the host plants were assessed by mixing with other ingredients composed of proteins, carbohydrates, minerals, and vitamins. The ratio of the ingredients was adopted from Nayar (1964) with slight modifications according to the various treatments as outlined in Table 1.

**Table 1.** Diet formulations tested for rearing *Ruspolia differens*

Treatments	Diet composition
Control (C)	Ground black soldier fly larvae (*Hermetia illucens* (L.)—30 g Soybean flour—30 g Maize flour—22 g Vitamin premix—4 g Ground cow bones—3.4 g
CD	Control diet + 22 g of dried and ground *Digitaria guyana*
CC	Control diet + 22 g of dried and ground *Cynadon dactylon*
CA	Control diet + 22 g of dried and ground *Ageratum conyzoides*
CM	Control diet + 22 g of dried and ground *Megathyrsus maximus*

The formulation was repeated as need arose. Ten *R. differens* nymphs (≤24 h old) were separately placed in 6 cm deep × 6 cm diameter plastic containers and replicated 4 times to make a total number of 40 insects per diet. The treatments were arranged in a Completely Randomized Design (CRD) under ambient conditions of 28 ± 1°C, 50–55% RH, and a photoperiod of 12:12 h L:D. The diets were offered *ad libitum* to all insects and were refreshed after 3 d. Individual insects were monitored daily until senescence. Live weights of adults were measured within 24 h after molting (Malinga et al. 2018a). Nymphal developmental durations were computed.

To determine adult fecundity, all adult females which reached adult stage were paired with males from the same diet. When the male died before the female, it was replaced with another male from the same diet or the main colony in case there were no more males from the same diet (Azrag et al. 2017). In all treatments, a small ball of moist cotton wool (approximately 50 mm diameter) was provided as a source of water and an oviposition substrate for female *R. differens*. Additionally, a stem of *M. maximus* was provided in each experimental unit as an oviposition substrate. Eggs were collected by opening the leaf sheath of grasses and by detaching them from inside and outside the cotton wool. Mortality was determined by considering the initial number of nymphs and the number of insects that reached the adult stage in each treatment.

### Data Analysis

Data were analyzed in R software (version 3.3.0) (R Core Team 2016) via the interface R studio (version 1.2.5). Data on *R. differens* development time, adult longevity, and fecundity were subjected to a log-linked poisson distribution. Over dispersion of data was assessed using the ratio of residual deviances to degrees of freedom; and where detected, it was corrected by fitting a negative binomial GLM-nb.glm using the MASS package (Venables and Ripley 2002). Mortality data were analyzed by logit-linked binomial GLM. Tukey’s test was used in pairwise multiple mean separation among treatments using *emmeans* package (Lenth and Lenth 2018).

Data on *R. differens* adult weight were normally distributed (Shapiro-Wilk test: *P* > 0.05) and their variances were homogenous (Levene’s test: *P* > 0.05). Therefore, these data were subjected to analysis of variance (ANOVA), and posthoc mean separation was performed using the Student–Newman–Keuls (SNK) test using the package “agricolae” (de Mendiburu 2020). All the statistical significances were determined at *P* < 0.05.

## Results

### Development of *R. differens* on Different Diets

**Fig. 2.**
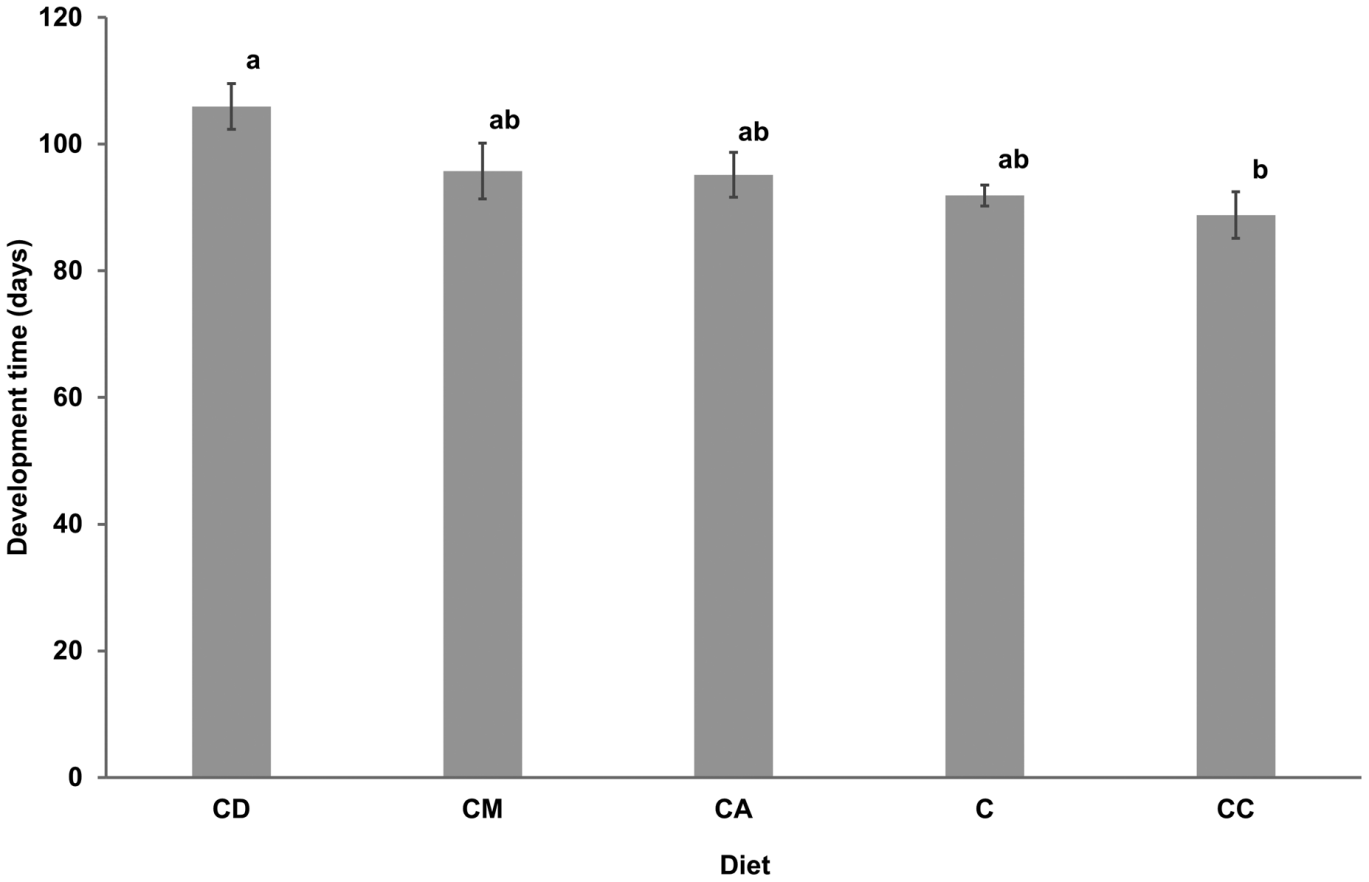
Mean developmental times of *Ruspolia differens* nymphs on different diets. Bars with the same letters are not significantly different (α = 0.05). Error bars represent standard errors of the mean. C = control diet; and CD, CC, CA, and CM = diet containing ingredients in the control diet plus *Digitria gayana*, *Cynodon dactylon*, *Ageratum conyzoides*, and *Megathyrsus maximus,* respectively.

There was a significant effect of mixing host plants with artificial diet on nymphal development time (χ ^2^ = 92.2, df = 87, *P* = 0.020). The longest nymphal development of 105.9 ± 3.6 d (range from 79 to 140 d) was recorded on the diet mixed with *D. gayana* while the shortest development time of 88.8 ± 3.7 d (range from 63 to 116 d) was recorded in the diet mixed with *C. dactylon* (Fig. 2). The significant difference was between diets mixed with *D. gayana* and that mixed with *C. dactylon*.

### Mortality of *R. differens* on Different Diets

**Fig. 3.**
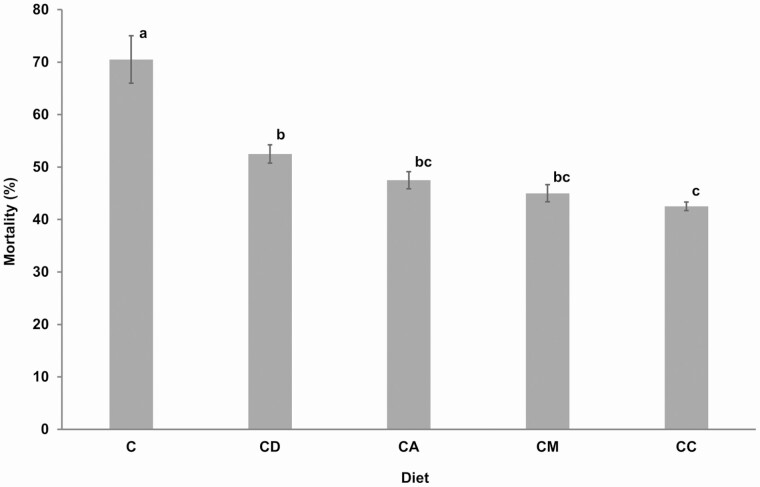
Mean mortality of *Ruspolia differens* on different diets. Bars with the same letters are not significantly different (α = 0.05). Error bars represent standard errors of the mean. C = control diet; and CD, CC, CA, and CM = diet containing ingredients in the control diet plus *Digitria gayana*, *Cynodon dactylon*, *Ageratum conyzoides*, and *Megathyrsus maximus,* respectively.

Incorporation of host plants into artificial diet significantly affected mortalities of *R. differens* (χ ^2^ = 30.87, df = 15, *P* < 0.001). The highest mortality of 70.5 ± 2.3% was recorded in the control diet while the lowest mortality of 42.5 ± 0.4% was recorded in *C. dactylon* incorporated diet (Fig. 3). A significantly higher mortality was observed between the control diet and all other diets with host plants. Apart from the control diet, significant difference was also observed between diets containing *D. gayana* and *C. dactylon*.

### Adult Longevity of *R. differens* in Diets Mixed with Host Plants

**Fig. 4.**
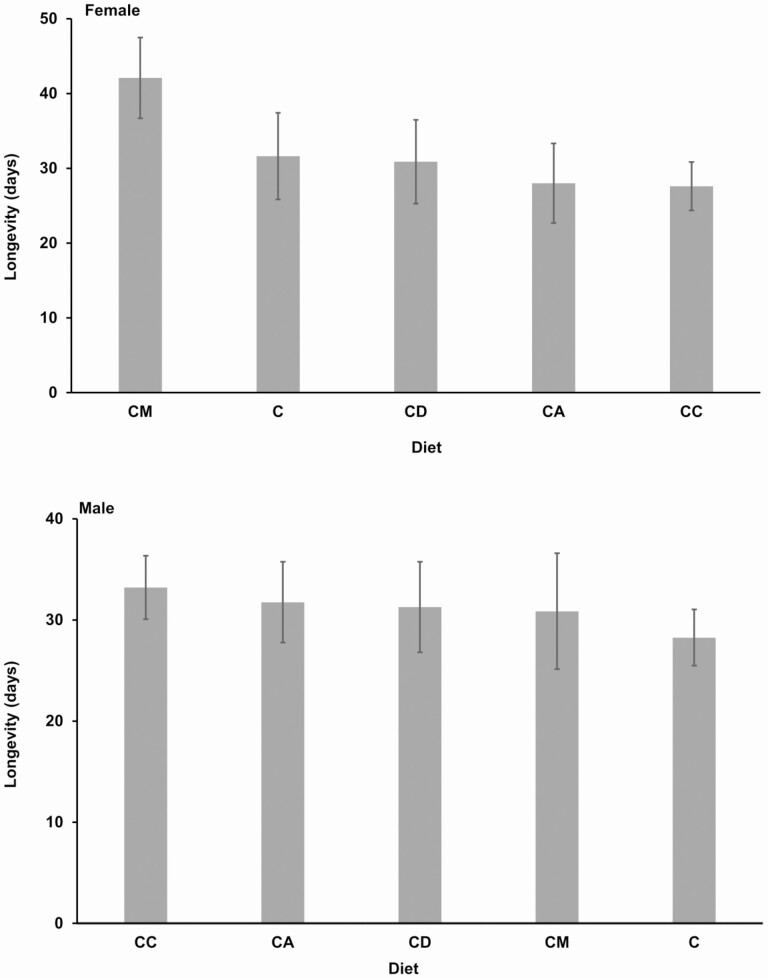
Mean adult longevity of *Ruspolia differens* on different diets. Error bars represent standard errors of the mean. C = control diet; and CD, CC, CA, and CM = diet containing ingredients in the control diet plus *Digitria gayana*, *Cynodon dactylon*, *Ageratum conyzoides*, and *Megathyrsus maximus,* respectively.

There was no significant effect of inclusion of host plants in the diet on longevity of both male and female *R. differens* (Females: χ ^2^ = 50.06, df = 44, *P* = 0.156, Males: χ ^2^ = 40.4, df = 35, *P* = 0.965) (Fig. 4).

### Fecundity of *R. differens* on Different Diets

**Fig. 5.**
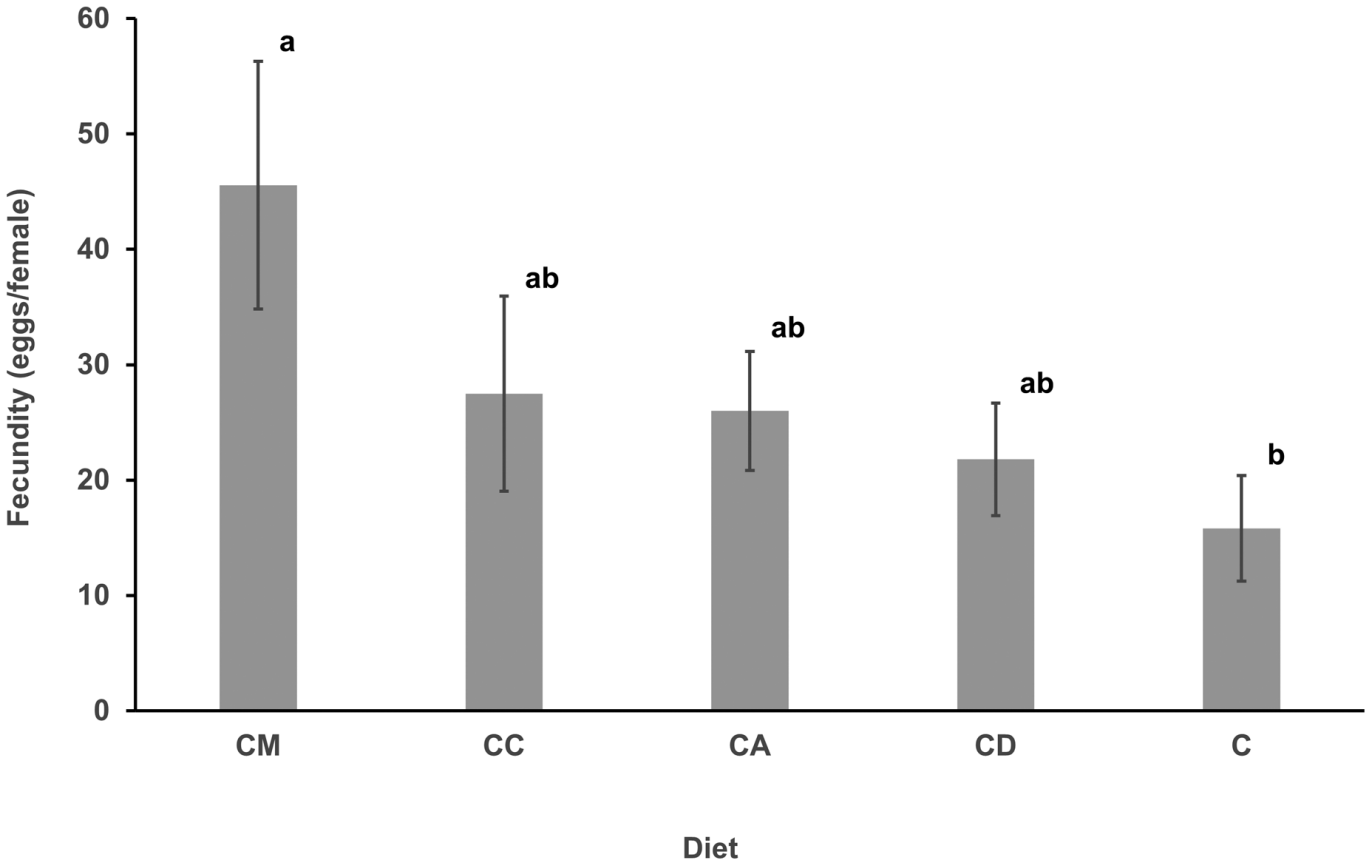
Mean fecundity of *Ruspolia differens* on different host plant diets. Bars with the same letters are not significantly different (α = 0.05). Error bars represent standard errors of the mean. C = control diet; and CD, CC, CA, and CM = diet containing ingredients in the control diet plus *Digitria gayana*, *Cynodon dactylon*, *Ageratum conyzoides*, and *Megathyrsus maximus,* respectively.

Host plant diets significantly affected the fecundity of *R. differens* (χ ^2^ = 36.79, df = 30, *P* = 0.005) (Fig. 5). The highest fecundity of 45.6 ± 10.7 eggs (range 31 to 95 eggs) was recorded in *M. maximus* based diet while the lowest fecundity of 15.8 ± 4.6 eggs (range 5 to 32 eggs) was recorded in the control diet. A significant difference was observed between *M. maximus* mixed diet and the control diet.

### Adult Weight of *R. differens* on Different Diets

**Fig. 6.**
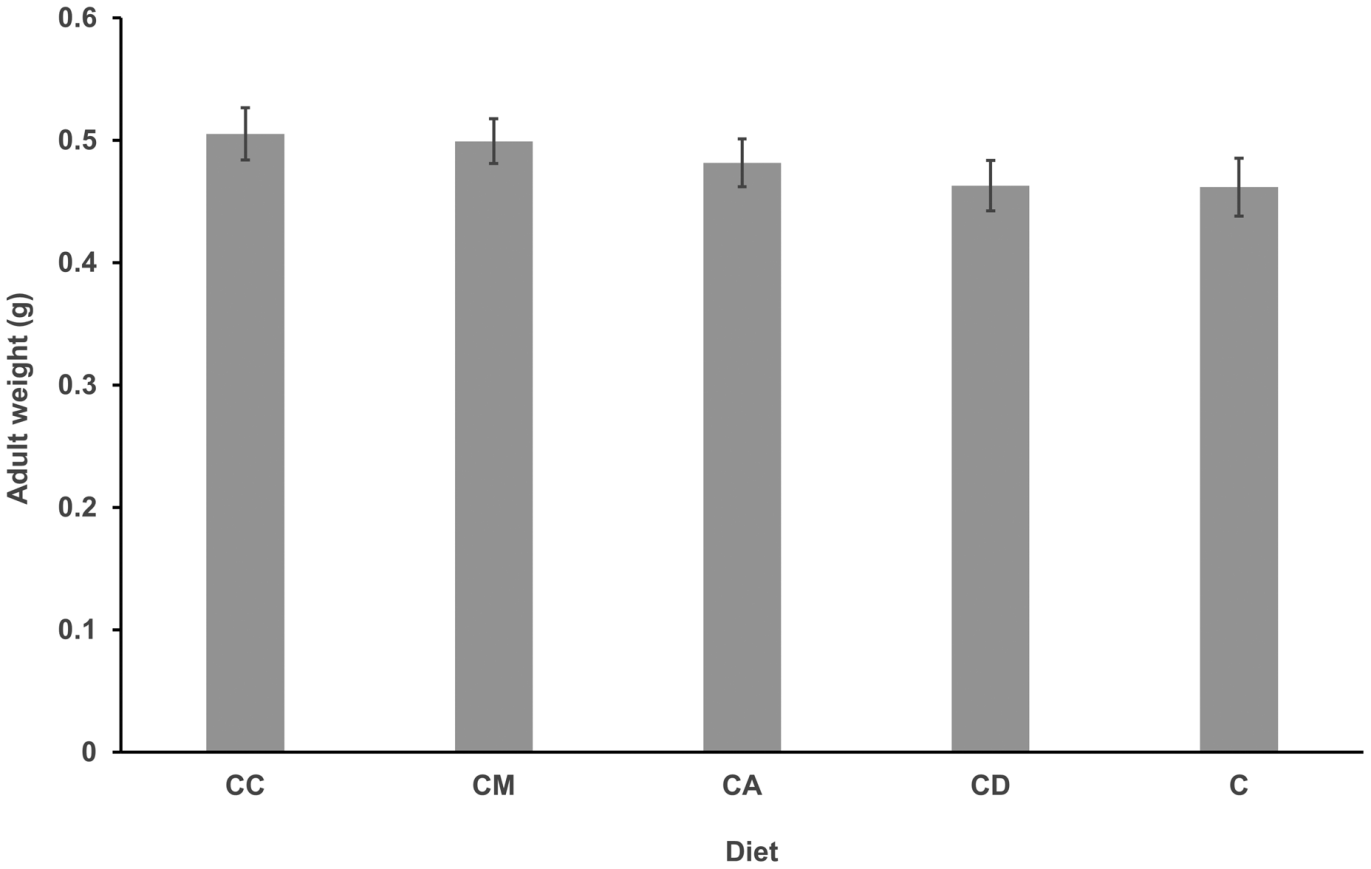
Mean adult weight of *Ruspolia differens* on different diets. Error bars represent standard errors of the mean. C = control diet; and CD, CC, CA, and CM = diet containing ingredients in the control diet plus *Digitria gayana*, *Cynodon dactylon*, *Ageratum conyzoides*, and *Megathyrsus maximus,* respectively.

There was no significant difference in *R. differens* adult weight among different host plant diets (F_4,92_ = 0.920, *P* = 0.450), ranging from 0.46 ± 0.02 g on the control to 0.51 ± 0.02 g (range 0.30 to 0.68g) on *C. dactylon* (Fig. 6).

## Discussion

Finding a solution to low survival of *R. differens* during artificial mass rearing remains a subject of intensive research (Brits and Thornton 1981, Malinga et al. 2018a). In this regard, factors that influence development, survival, and reproduction of *R. differens* under captive mass rearing such as optimum temperatures, host plants, and oviposition substrates have been determined (Lehtovaara et al. 2019, Opoke et al. 2019, Leonard et al. 2020, Egonyu et al. 2021, Leonard et al. 2021). Despite all these efforts, more work remains to be done to develop protocols for efficient mass production of the insect. For instance, although host plants of *R. differens* have been determined, effect of their inclusion in the diets on the performance of the insect has not yet been investigated.

In this study, we recorded the shortest *R. differens* development in the diet mixed with *C. dactylon*; while the longest development time was recorded in *D. gayana* based diet. The difference in development time recorded in different hosts could be attributed to naturally occurring chemical components of plants that vary greatly across species (Blanco et al. 2009). Addition of some naturally occurring plant components such as gossypol and nicotine to artificial diets negatively influences development in *Chloridea* (=*Heliothis*) *virescens* (F.) (Lepidoptera: Noctuidae) and *Campoletis sonorensis* (Cameron) (Hymenoptera: Ichneumonidae) (Gunasena et al. 1988, Gunasena et al. 1990, Blanco et al. 2009). The gossypol and nicotine are mainly found in cotton and tobacco, respectively (Gunasena et al. 1990, Blanco et al. 2009). The plant components which might have influenced *R. differens* development time in this study ought to be elucidated. Behmer and Elias (2000) reported that five species of acridid grasshoppers developed optimally on diets containing 28% protein, 28% digestible carbohydrate, 39.7% cellulose, 2.4% minerals, 0.5% linoleic acid, 0.3% ascorbic acid, 0.2% vitamin, and 0.2% phenylalanine. Further probing is required to determine whether this proposition holds for grasshoppers from other families such Tettigoniidae.

Nutrient-deficient diets have been reported to prolong the development time of grasshoppers including *R. differens* (Bernays and Bright 1991, Miura and Ohsaki 2004, Malinga et al. 2018a). The development times of *R. differens* in all diet treatments in this study were shorter than 150 d reported by Malinga et al. (2018a) in a single diet, but partially comparable to 100 d in the mixed diet of six and eight plant and nonplant food components. The enhanced development in the current study could be attributed to inclusion of black soldier fly and soybean meals as protein-rich supplements which reportedly boost insect development rates (Yamka et al. 2003, Berner et al. 2005, Tschirner and Simon 2015, Shumo et al. 2019).

Our data demonstrate a significantly lower mortality of *R. differens* (42.5–52.5%) in all diets mixed with host plants compared to the control diet which recorded over 70% mortality. The high survivorship in the host plant-based diet could be attributed to occurrence of aromatic plant amino acids such as phenylalanine which is essential for cuticle composition (Behmer and Joern 1993). However, chemical analysis of the plants used in feeding *R. differens* in the current study is required to verify contents of essential nutrients.

The lowest mortality (42.5%) of *R. differens* in diets mixed with host plants was lower than the lowest mortality of 46.7% reported by Ssepuuya et al. (2018) in *R. differens* reared on single live plants of *C. dactylon* and *M. maximus*. The mortality of *R. differens* in this study was also lower than the average mortality of 61.9% reported by Malinga et al. (2018a) in the diet of two (rice seed heads and finger millet seed heads) and eight (rice seed heads, finger millet seed heads, wheat bran, chicken superfeed egg booster, sorghum seed heads, germinated finger millet, simsim cake, and dog biscuit pellets) components. Foieri et al. (2020) also recorded high influence of host plants (Poaceae) on survival of *Notozulia entreriana* Berg (Hemiptera: Cercopidae), whereby 2.5, 22.5, and 95% survivals were recorded when the nymphs were reared on *Zea mays* L. (Poales: Poaceae), *Chloris gayana* Kunth (Poales: Poaceae), and *Brachiaria decumbens* Stapf (Poales: Poaceae), respectively. Improved *R. differens* survival in this study could be due to high carbohydrate levels from maize flour. Joern and Behmer (1997) reported that the white-whiskered grasshopper *Ageneotettix deorum* Scudder (Orthoptera: Acrididae) reared on the diet with 26.7% carbohydrates survived for 50.2 d but could only survive for 28.5 d on the diet comprising 4.3% carbohydrate. Also, survival differences in insects reared on different host plants could be attributed to nitrogen contents in host plants (Berner et al. 2005). Some insects such as grasshoppers can balance nitrogen levels in their body by compensatory feeding or through selecting host plants with high nitrogen and carbohydrate contents (Berner et al. 2005, Mayntz et al. 2005). Their strong chewing mouth parts (Matojo and Hosea 2013) could facilitate their wide selection of nutrient-rich host plants.

The highest and lowest *R. differens* fecundity were recorded in *M. maximus* and the control diets, respectively. Differences in fecundity among the treatments could be attributed to the concentration of amino acids and sterols in host plants (Awmack and Leather 2002). Phloem amino acid concentration has been reported to influence the fecundity of aphids (*Myzus persicae* Sulzer [Hemiptera: Aphididae]) in potatoes. Low levels of plant sterols in artificial diets decreased fecundity of the diamondback moth *Plutella xylostella* Linnaeus (Lepidoptera: Plutellidae). It is notable that the fecundity of *R. differens* (21.8 to 45.5 eggs/female) in this study was markedly lower than the average fecundity of ~188 eggs reported by Leonard et al. (2021) under variable temperatures and diet composition. The current finding also disagrees with other studies by Brits and Thornton (1981) and Malinga et al. (2018a) who reported *R. differens* fecundity of 257 eggs and 148 eggs, respectively. The differences in *R. differens* fecundity between these studies could be attributed to the diet composition; as the diets by Malinga et al. (2018a) and Brits and Thornton (1981) comprised live plant seed heads, balanced protein, and formulated chicken and dog food, while our study involved diets containing powders of dry host plants, protein, carbohydrate, minerals, and vitamins. The dried host plant diets used in the current study would be preferable for storage of feed under a commercial mass production, but the effect of drying on host plant chemistry which may influence fecundity in the insect should be investigated. Nutritional requirements of nymphal and adult stages can also influence the fecundity of insects (Adams 2000, Wittmeyer et al. 2001). The nutritional requirements of *R. differens* nymphal and adult stages have not yet been investigated, hence further research is warranted to elucidate this.

The results show that inclusion of host plants in diets had no effect on longevity of *R. differens*. The adult longevity across the diets ranged from about 31 to 42 d, which closely concurs with the finding by Leonard et al. (2021) in which maximum *R. differens* adult longevity of 39 d was recorded. However, the *R. differens* adult longevity in the current study was much shorter than the highest female longevity of 72 d and 88 d reported by Brits and Thornton (1981) and Malinga et al. (2018a), respectively. The contradicting adult longevities could be contributed by the levels of protein in diets (Cammack and Tomberlin 2017). Runagall-McNaull et al. (2015) reported that whereas the banana stalk fly, *Derocephalus angusticollis* Enderlein (Diptera: Neriidae) lived for 97.7 d on the diet containing moderate protein level of 11 g/L, its lifespan was only 56.5 d on the diet with low (3 g/L) or high (30 g/L) protein content.

Inclusion of host plants in diets had no significant effect on *R. differens* adult weigh, which ranged from 0.46 ± 0.02 g to 0.51 ± 0.02 g. This range closely corroborates the finding by Ssepuuya et al. (2018) that the highest *R. differens* adult weight, when reared on *Eleusine indica* L. (Poales: Poaceae), was 0.586 g. However, Ssepuuya et al. (2018) found that *R. differens* adult weight varied significantly when reared on live *C. dactylon*, *E. indica*, *M. maximus*, and a combination of the three plants. In this study, protein was supplied by black soldier fly and soybean flour while the study by Ssepuuya et al. (2018) included no other protein sources besides the live plants.

### Conclusion

This study demonstrates that some natural host plants of *R. differens* identified from the guts of wild conspecific are critical in the development, survival, and reproduction of the insects and therefore should be included in diet formulations for its artificial mass rearing. Further analysis of the host plants to determine chemical components facilitating these favorable attributes of the insects during rearing is required.

## References

[CIT0001] Adams, T. S . 2000. Effect of diet and mating status on ovarian development in a predaceous stink bug *Perillus bioculatus* (Hemiptera: Pentatomidae). Ann. Entomol. Soc. Am. 93: 529–535.

[CIT0002] Agea, J. G., D. Biryomumaisho, M. Buyinza, and G. N. Nabanoga. 2008. Commercialization of *Ruspolia nitidula* (nsenene grasshoppers) in central Uganda. Afr. J. Food Agric. Nutr. Dev. 8: 319–332.

[CIT0003] Awmack, C. S., and S. R. Leather. 2002. Host plant quality and fecundity in herbivorous insects. Annu. Rev. Entomol. 47: 817–844. 11729092 10.1146/annurev.ento.47.091201.145300

[CIT0004] Azrag, A. G., L. K. Murungi, H. E. Tonnang, D. Mwenda, and R. Babin. 2017. Temperature-dependent models of development and survival of an insect pest of African tropical highlands, the coffee antestia bug *Antestiopsis thunbergii* (Hemiptera: Pentatomidae). J. Therm. Biol. 70: 27–36. 29108555 10.1016/j.jtherbio.2017.10.009

[CIT0005] Bailey, W. J., and A. W. R. McCrae. 1978. The general biology and phenology of swarming in the East African tettigoniid *Ruspolia differens* (Serville) (Orthoptera). J. Nat. Hist. 12: 259–288.

[CIT0006] Behmer, S. T., and D. O. Elias. 2000. Sterol metabolic constraints as a factor contributing to the maintenance of diet mixing in grasshoppers (Orthoptera: Acrididae). Physiol. Biochem. Zool. 73: 219–230. 10801400 10.1086/316728

[CIT0007] Behmer, S. T., and A. Joern. 1993. Diet choice by a grass-feeding grasshopper based on the need for a limiting nutrient. Func. Ecol. 7: 522–527.

[CIT0008] Bernays, E. A., and K. L. Bright. 1991. Dietary mixing in grasshoppers: switching induced by nutritional imbalances in foods. Entomol. Exp. Appl. 61: 247–253.

[CIT0009] Bernays, E. A., K. L. Bright, N. Gonzalez, and J. Angel. 1994. Dietary mixing in a generalist herbivore: tests of two hypotheses. Ecology. 75: 1997–2006.

[CIT0010] Berner, D., W. U. Blanckenhorn, and C. Körner. 2005. Grasshoppers cope with low host plant quality by compensatory feeding and food selection: N limitation challenged. Oikos. 111: 525–533.

[CIT0011] Blanco, C. A., M. Portilla, C. A. Abel, H. Winters, R. D. Ford, and D. Streett. 2009. Soybean flower and wheat germ proportions in artificial diet and their effect on the growth rates of the tobacco budworm, *Heliothis virescen*s. J. Insect Sci. 9: 1–9. 10.1673/031.009.5901 PMC3011917 20050778

[CIT0012] Brits, J. A., and C. H. Thornton. 1981. On the biology of *Ruspolia differens* (Serville) (Orthoptera: Tettigoniidae) in South Africa. Phytophylactica 13: 169–174.

[CIT0013] Cammack, J. A., and J. K. Tomberlin. 2017. The impact of diet protein and carbohydrate on select life-history traits of the black soldier fly *Hermetia illucens* (L.)(Diptera: Stratiomyidae). Insects. 8: 56. 10.3390/insects8020056 PMC5492070 28561763

[CIT0014] Egonyu, J. P., M. M. Miti, C. M. Tanga, L. Alfonce, and S. S. Subramanian. 2021. Cannibalism, oviposition and egg development in the edible long-horned grasshopper, *Ruspolia differens* (Orthoptera Tettigoniidae) under laboratory conditions. J. Insects Food Feed. 7: 89–97.

[CIT0015] Foieri, A., E. G. Virla, A. Maciá, and A. M. Marino de Remes Lenicov. 2020. Effect of host plant on the fitness of the spittlebug *Notozulia entreriana*: alternative method for rearing. Entomol. Exp. Appl. 168: 618–625.

[CIT0016] Gunasena, G. H., S. B. Vinson, H. J. Williams, and R. D. Stipanovic. 1988. Effects of caryophyllene, caryophyllene oxide, and their interaction with gossypol on the growth and development of *Heliothis virescens* (F.) (Lepidoptera: Noctuidae). J. Econ. Entomol. 81: 93–97.

[CIT0017] Gunasena, G. H., S. B. Vinson, and H. J. Williams. 1990. Effects of nicotine on growth, development, and survival of the tobacco budworm (Lepidoptera: Noctuidae) and the parasitoid *Campoletis sonorensis* (Hymenoptera: Ichneumonidae). J. Econ. Entomol. 83: 1777–1782.

[CIT0018] Hartley, J. C . 1967. Laboratory culture of a Tettigoniid, *Homorocoryphus nitidulus* vicinus (Wlk.) (Orthoptera). Bull. Entomol. Res. 57: 203–205.

[CIT0019] Joern, A., and S. T. Behmer. 1997. Importance of dietary nitrogen and carbohydrates to survival, growth, and reproduction in adults of the grasshopper *Ageneotettix deorum* (Orthoptera: Acrididae). Oecologia. 112: 201–208. 28307571 10.1007/s004420050301

[CIT0020] Joern, A., and S. T. Behmer. 1998. Impact of diet quality on demographic attributes in adult grasshoppers and the nitrogen limitation hypothesis. Ecol. Entomol. 23: 174–184.

[CIT0021] Kinyuru J. N. , G. M. Kenji, S. N. Muhoho, and M. Ayieko. 2010. Nutritional potential of longhorn grasshopper (*Ruspolia differens*) consumed in Siaya district, Kenya. J. Agr. Sci. Tech. 12: 32–46.

[CIT0022] Kinyuru, J. N., D. Nyangena, E. Kamau, A. Nderitu, J. Muniu, C. Kipkoech, J. Weru, N. Ndung’u, and M. Mmari. 2018. The role of edible insects in diets and nutrition in East Africa, pp. 93–108. *In* A. Halloran et al. (eds.), Edible insects in sustainable food systems. Springer, New York, NY.

[CIT0023] Lattanzio, V., V. M. Lattanzio, and A. Cardinali. 2006. Role of phenolics in the resistance mechanisms of plants against fungal pathogens and insects. Adv. Res. 661: 23–67.

[CIT0024] Lehtovaara, V. J., H. Roininen, and A. Valtonen. 2018. Optimal temperature for rearing the edible *Ruspolia differens* (Orthoptera: Tettigoniidae). J. Econ. Entomol. 111: 2652–2659. 30124900 10.1093/jee/toy234

[CIT0025] Lehtovaara, V. J., J. Tahvanainen, J. Sorjonen, A. Valtonen, and H. Roininen. 2019. Space and shelter requirement of nymphs in the mass-rearing of the edible *Ruspolia differens* (Orthoptera: Tettigoniidae). J. Econ. Entomol. 112: 1651–1657. 30937445 10.1093/jee/toz065

[CIT0026] Lenth, R., and M. R. Lenth. 2018. Package ‘lsmeans’. Am. Stat. 34: 216–221.

[CIT0027] Leonard, A., F. M. Khamis, J. P. Egonyu, S. Kyamanywa, S. Ekesi, C. M. Tanga, R. S. Copeland, and S. Subramanian. 2020. Identification of edible short-and long-horned grasshoppers and their host plants in East Africa. J. Econ. Entomol. 113: 2150–2162. 33063829 10.1093/jee/toaa166

[CIT0028] Leonard, A., J. P. Egonyu, C. M. Tanga, S. Kyamanywa, H. Z. Tonnang, A. G. Azrag, F. M. Khamis, S. Ekesi, and S. Subramanian. 2021. Predicting the current and future distribution of the edible long-horned grasshopper *Ruspolia differens* (Serville) using temperature-dependent phenology models. J. Therm. Biol. 95: 102786. 33454030 10.1016/j.jtherbio.2020.102786

[CIT0029] Malinga, G. M., A. Valtonen, V. J. Lehtovaara, K. Rutaro, R. Opoke, P. Nyeko, and H. Roininen. 2018a. Mixed artificial diets enhance the developmental and reproductive performance of the edible grasshopper, *Ruspolia differens* (Orthoptera: Tettigoniidae). Appl. Entomol. Zool. 53: 237–242.

[CIT0030] Malinga, G. M., A. Valtonen, V. J. Lehtovaara, K. Rutaro, R. Opoke, P. Nyeko, and H. Roininen. 2018b. Diet acceptance and preference of the edible grasshopper *Ruspolia differens* (Orthoptera: Tettigoniidae). Appl. Entomol. Zool. 53: 229–236.

[CIT0031] de Mandimburu, F . 2020. agricolae: Statistical Procedure for Agricultural Research. R package version 1.3–2. https://CRAN.R-project.org/package=agricolae. Accessed on May 20, 2020.

[CIT0032] Matojo, D. N. and K. M. Hosea. 2013. Phylogenetic Relationship of the Long-horned Grasshopper *Ruspolia differens* Serville (Orthoptera: Tettigoniidae) from northwest Tanzania based on 18S ribosomal nuclear sequences. J. Insects. Article id 504285, 5 p.

[CIT0033] Massa, B . 2015. Taxonomy and distribution of some katydids (Orthoptera: Tettigoniidae) from tropical Africa. ZooKeys. 524: 17–44. 10.3897/zookeys.524.5990 PMC4602289 26478704

[CIT0034] Mayntz, D., D. Raubenheimer, M. Salomon, S. Toft, and S. J. Simpson. 2005. Nutrient-specific foraging in invertebrate predators. Science. 307: 111–113. 15637278 10.1126/science.1105493

[CIT0035] Miura, K., and N. Ohsaki. 2004. Diet mixing and its effect on polyphagous grasshopper nymphs. Ecol. Res. 19: 269–274.

[CIT0036] Mmari, M. W., J. N. Kinyuru, H. S. Laswai, and J. K. Okoth. 2017. Traditions, beliefs and indigenous technologies in connection with the edible longhorn grasshopper *Ruspolia differens* (Serville 1838) in Tanzania. J. Ethnobiol. Ethnomedicine. 13: 60. 10.1186/s13002-017-0191-6 PMC5683242 29132398

[CIT0038] Nayar, J. K . 1964. The nutritional requirements of grasshoppers: I. rearing of the grasshopper, *Melanoplus bivittatus* (Say), on a completely defined synthetic diet and some effects of different concentrations of b-vitamin mixture, linoleic acid, and β-carotene. Can. J. Zool. 42: 11–22.

[CIT0039] Okia, C. A., W. Odongo, P. Nzabamwita, J. Ndimubandi, N. Nalika, and P. Nyeko. 2017. Local knowledge and practices on use and management of edible insects in Lake Victoria basin, East Africa. J. Insects as Food Feed. 3: 83–93.

[CIT0040] Opoke, R., P. Nyeko, G. M. Malinga, K. Rutaro, H. Roininen, and A. Valtonen. 2019. Host plants of the non-swarming edible bush cricket *Ruspolia differens*. Ecol. Evol. 9: 3899–3908. 31015975 10.1002/ece3.5016 PMC6467855

[CIT0041] Paul, A., M. Frederich, R. Uyttenbroeck, S. Hatt, P. Malik, S. Lebecque, M. Hamaidia, K. Miazek, D. Goffin, L. Willems, and M. Deleu. 2016. Grasshoppers as a food source? A review. Biotechnol. Agron. Soc. Environ. 20: 337–352.

[CIT0042] R Core Team . 2016. R: A language and environment for statistical computing. R Foundation for Statistical Computing, Vienna, Austria. https://www.R–project.org/

[CIT0043] Runagall-McNaull, A., R. Bonduriansky, and A. J. Crean. 2015. Dietary protein and lifespan across the metamorphic boundary: protein-restricted larvae develop into short-lived adults. Sci. Rep. 1: 1–7. 10.1038/srep11783 PMC4484247 26119686

[CIT0044] Rutaro, K., G. M. Malinga, V. J. Lehtovaara, R. Opoke, P. Nyeko, H. Roininen, and A. Valtonen. 2018. Fatty acid content and composition in edible *Ruspolia differens* feeding on mixtures of natural food plants. BMC Res. Notes. 11: 687. 30285897 10.1186/s13104-018-3792-9 PMC6167896

[CIT0045] Shumo, M., I. M. Osuga, F. M. Khamis, C. M. Tanga, K. K. Fiaboe, S. Subramanian, S. Ekesi, A. van Huis, and C. Borgemeister. 2019. The nutritive value of black soldier fly larvae reared on common organic waste streams in Kenya. Sci. Rep. 9: 1–13. 31300713 10.1038/s41598-019-46603-z PMC6626136

[CIT0046] Singer, M. S., and E. A. Bernays. 2003. Understanding omnivory through food mixing behavior. Ecology. 84: 2532–2537.

[CIT0047] Ssepuuya, G., C.M. Tanga, I. Yekko, F. Sengendo, C.T. Ndagire, K.K.M. Fiaboe, J. Karungi, and D. Nakimbugwe. 2018. Suitability of egg hatching conditions and commonly available food plants for rearing the long-horned grasshopper *Ruspolia differens* Serville (Orthoptera: Tettigoniidae). J. Insects Food Feed. 4: 253–261.

[CIT0048] Ssepuuya, G., R. Smets, D. Nakimbugwe, M. Van Der Borght, and J. Claes. 2019. Nutrient composition of the long-horned grasshopper *Ruspolia differens* Serville: effect of swarming season and sourcing geographical area. Food Chem. 301: 125305. 31387042 10.1016/j.foodchem.2019.125305

[CIT0049] Tschirner, M., and A. Simon. 2015. Influence of different growing substrates and processing on the nutrient composition of black soldier fly larvae destined for animal feed. J. Insects as Food Feed. 1: 249–259.

[CIT0050] Valtonen, A., G. M. Malinga, P. Junes, R. Opoke, V. J. Lehtovaara, P. Nyeko, and H. Roininen. 2018. The edible katydid *Ruspolia differens* is a selective feeder on the inflorescences and leaves of grass species. Entomol. Exp. Appl. 166: 592–602.

[CIT0051] Venables, W. N., and B. D. Ripley. 2002. Modern Applied Statistics with S. 4th ed. Springer, New York, USA.

[CIT0052] Wittmeyer, J. L., T. A. Coudron, and T. S. Adams. 2001. Ovarian development, fertility and fecundity in *Podisus maculiventris* Say (Heteroptera: Pentatomidae): an analysis of the impact of nymphal, adult, male and female nutritional source on reproduction. Invertebr. Reprod. Dev. 39: 9–20.

[CIT0053] Yamka, R. M., U. Jamikorn, A. D. True, and D. L. Harmon. 2003. Evaluation of soyabean meal as a protein source in canine foods. Anim. Feed Sci. Technol. 109: 121–132. 10.2527/2003.8192279x 12968703

